# Supplementary material for: A high-resolution mRNA expression time course of embryonic development in zebrafish
Source: eLife. 2017 Nov 16;6:e30860. doi: 10.7554/eLife.30860 (PMC5690287; doi:10.7554/eLife.30860)
Supplement: Supplementary file 6. [file elife-30860-supp6.zip › biolayout-clusters-files/Cluster237-genes.html]

Cluster237


# Cluster237: Genes

| Ensembl ID | Gene Name | Chr | Start | End | Biotype |
| --- | --- | --- | --- | --- | --- |
| ENSDARG00000001754 | noc2l | 23 | 23199579 | 23252866 | protein\_coding |
| ENSDARG00000020077 | nol10 | 17 | 51593394 | 51631768 | protein\_coding |
| ENSDARG00000087779 | pum3 | 10 | 15363383 | 15382271 | protein\_coding |
| ENSDARG00000025332 | rbm28 | 18 | 10724227 | 10744696 | protein\_coding |
| ENSDARG00000022410 | rrp12 | 13 | 31212876 | 31239734 | protein\_coding |
| ENSDARG00000020278 | si:dkeyp-120h9.1 | 19 | 32155791 | 32178302 | protein\_coding |
